# Psychological Impact of the COVID-19 Outbreak on Mental Health Outcomes among Youth: A Rapid Narrative Review

**DOI:** 10.3390/ijerph18116067

**Published:** 2021-06-04

**Authors:** Federica Cielo, Randi Ulberg, Dina Di Giacomo

**Affiliations:** 1 Department of Life, Health and Environmental Sciences, University of L’Aquila, 67100 L’Aquila, Italy; federica.cielo@graduate.univaq.it; 2 Postgraduate School on Clinical Psychology, University of L’Aquila, 67100 L’Aquila, Italy; 3 Division of Mental Health Addiction, University of Oslo, 0319 Oslo, Norway; randi.ulberg@medisin.uio.no; 4 Department of Psychiatry, Diakonhjemmet Hospital, 0319 Oslo, Norway

**Keywords:** COVID-19, pandemic, psychological impact, mental health, young

## Abstract

The coronavirus disease (COVID-19) outbreak has affected not only physical health but also mental health and psychological wellbeing. This narrative review aimed to map the literature on the psychological impact on the young generation of the COVID-19 pandemic, social restrictions, and extraordinary measures to curb the spread of coronavirus. We performed a systematic search of MEDLINE through PubMed and Web of Science [Science Citation Index Expanded, SCI-EXPANDED), Social Sciences Citation Index (SSCI), and Emerging Sources Citation Index (ESCI)] of all scientific literature published from May 2020 until 15 March 2021. Based on inclusion and exclusion criteria, a total of 15 articles were included. We conducted a narrative review. The reviewed articles suggested the impact of the pandemic and lockdown measures on young persons for several mental symptoms as well as anxiety, stress, depression, event-specific distress, decrease in psychological wellbeing, and changes in sleep habits. Psychological symptoms were related to the experience of several stressors, such as risk for reduction of academic perspectives, massive e-learning adoption, economic issues, social restrictions, and implications for daily life related to the COVID-19 outbreak. This narrative review points out the negative psychological impact of the pandemic outbreak and the high vulnerability of the young in the development of psychological distress, highlighting the relevant focus on the mental health of young people during the pandemic and the need for structured and tailored psychological support and interventions focused to the improvement of Quality of Life of university students after the pandemic experience.

## 1. Introduction

The coronavirus disease 2019 (COVID-19), is a highly infectious disease often including severe acute respiratory syndrome. Coronavirus 2 (SARS-CoV-2) was first identified in Wuhan, Hubei Province, China in December 2019 [1]. In the following months, infection spread across other countries around the world as an emerging global health threat. In March 2020, the World Health Organization (WHO) declared it a pandemic [2]. The clinical features of COVID-19 are varied, ranging from asymptomatic states to acute respiratory distress syndrome and multi-organ dysfunction. Human-to-human transmission occurs through common pathways such as direct transmission, contact transmission, and airborne transmission: cough, sneeze, droplet inhalation, contact with oral, nasal, and eye mucous membranes are the common modes of spread [3]. COVID-19 provoked significant challenges to curb the spread of the infection and maintain global health security. Due to the rapid spread of the coronavirus, many countries implemented a range of anti-epidemic measures, such as keeping physical distance, wearing face masks, quarantine, and lockdown restrictions to contain the transmission and avoid contact with others. The pandemic has had a devastating impact on the global economy and the health of communities across the world [4]. Furthermore, the COVID-19 outbreak has affected not only physical health but also mental health and wellbeing [5]. The COVID-19 pandemic has severely affected the entire general population; even young people have not been spared from the changes of this unprecedented situation. Research on previous epidemic situations, including those of the acute respiratory syndrome (SARS) [6,7,8,9,10], the 2009 novel influenza A (H1N1) [11,12], and the 2018 Ebola pandemic [13], already revealed side effects on mental health. Based on these previous findings, researchers conducted several studies to investigate the consequences of the COVID-19 pandemic on mental health in specific groups or in the general population e.g., health workers, women, children, and young adults [14,15,16,17,18,19,20,21].

The present study aimed to review the emerging literature on COVID-19 to investigate the psychological impact of the COVID-19 outbreak, lockdown restriction, and extraordinary measures to curb the spread of coronavirus on mental health outcomes among youth. Further, we wanted to analyze COVID-19 related risk factors in order to capture potentially stressful events related to the increase of coronavirus spread out.

## 2. Methods

We conducted descriptive analyses (frequencies, incidence, percentage) to map the literature for the present narrative review on the psychological impact of the COVID-19 outbreak among youth. The framework outlined by Arksey and O’Malley [22] in their methodological paper was used. Our aim was to synthesize current available scientific literature related to the impact of mental health aspects on young people related to the COVID-19 outbreak and lockdown conditions.

### 2.1. Search Strategy

To identify potentially relevant studies for inclusion, we performed a systematic search of MEDLINE through PubMed and Web of Science [Science Citation Index Expanded (SCI-EXPANDED), Social Sciences Citation Index (SSCI), Emerging Sources Citation Index (ESCI)] in March 2021, with the terms ‘covid-19′, ‘psychological impact’, ‘mental health disorders’, ‘lockdown’ and ‘young’. We limited our search from May 2020 to March 2021. The last search was run on 15 March 2021. In addition, we consulted all citations of eligible articles for supplementary references that were missed in the initial search.

### 2.2. Inclusion and Exclusion Criteria

We included all papers related to the psychological impact of the COVID-19 outbreak published in English until 15 March 2021 related to young adults. However, we excluded reports that were not published in scientific peer-reviewed journals, reviews and studies in which the sample target (age range 18–30 years old) was not young people.

### 2.3. Article Selection and Data Extraction

To ensure the reliability of the narrative review, two reviewers independently screened for inclusion all publication titles and abstract. The following information was extracted from each paper: (a) authors, (b) title, (c) source title, (d) publication year, (e) age range, (f) topic (COVID-19, coronavirus, mental health).

### 2.4. Statistical Analysis

We conducted a descriptive analysis of the characteristics of the included literature to examine the psychological impact of the COVID-19 outbreak among young people. We conducted this narrative review according to the PRISMA Flow Diagram [23].

## 3. Results

### 3.1. Search Results

The literature search of PubMed and Web of Science database provided a total of 214 publications. After removing duplicates, 179 papers were identified for screening. Based on the criteria, two reviewers screened for inclusion all publication titles and abstracts, and successfully resolved all disagreements by consensus. Of these, we excluded records that were clearly off-topic papers; reviews; and articles only focused on psychiatric patients, patients with medical co-morbidity, pregnant women, community members, athletes, health workers, children, and adults. Furthermore, we excluded the general population because, even if it considered our reference target, it also included non-interest sample target for the purposes of our review. The full texts of the remaining 20 citations were examined comprehensively and identified as potentially eligible articles. An additional eight studies that met criteria for inclusion were identified by checking all citations of eligible articles for supplementary references that were missed in the initial search. Of these eight additional records from other sources, three were selected. Out of a total of 28 articles assessed for eligibility, 13 studies did not meet the inclusion criteria. The population investigated in these excluded papers, included individuals other than young people in the age range 18–30 years old. Finally, 15 articles were included in the present narrative review.

**Figure 1:**
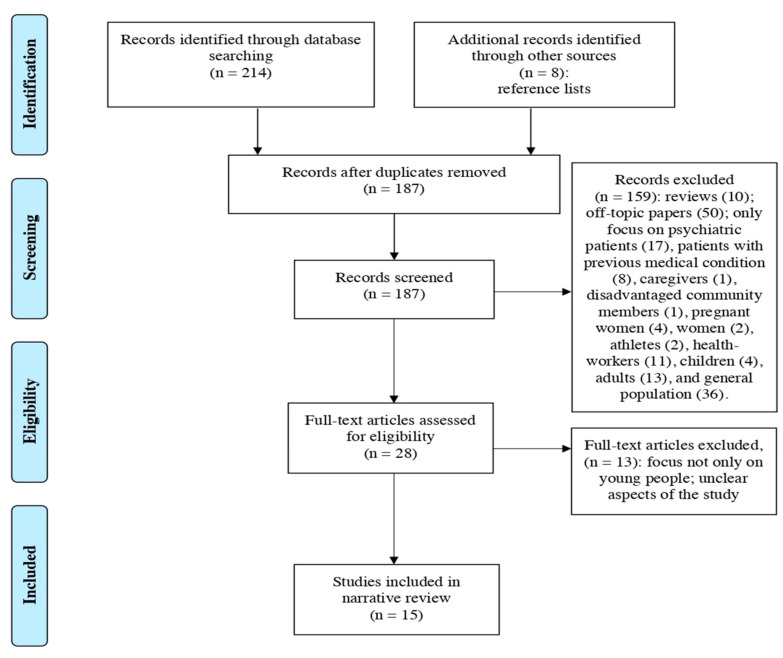
PRISMA Flowchart of the studies selection process for the narrative review on the psychological impact of the COVID-19 outbreak among youth.

Figure 1 illustrates the study selection flowchart.

### 3.2. Characteristics of Included Articles

Table 1 synthesizes the main characteristics of the included studies. We extracted from each included paper: (a) study design, (b) authors, (c) sample size, (d) age range, (e) sample, (f) data collection timing, and (g) recruitment. All the papers were grouped according to the study design (cross-sectional study and longitudinal study) and include young people as participants. Regarding the targeted sample, we observed that all studies concerned college or university students except two [24,25], which focused on young workers and young in a lockdown condition. Furthermore, according to the pandemic situation, lockdown restriction, and extraordinary measures to curb the spread of the coronavirus, almost all of the studies collected data through online survey. Generally, the survey link was disseminated in various platforms available on social media (e.g., Facebook, WhatsApp, email invitation, Google Forms). Only one study [26] did not mention the recruitment method. Most recruiting was conducted by convenience sampling, bypassing the probability sampling method: this procedure has a relevant risk of sampling error and lack of representation of the population, which might affect the finding. Nevertheless, the extraordinary pandemic condition needed to be considered.

Regarding the research design, the articles included in the present narrative review were 12 cross-sectional studies [26,27,28,29,30,31,32,33,34,35,36,37] and three longitudinal studies [24,25,38].

Overall, selected studies involved 18,495 participants, and the recruiting lasted from 1 to 2 months.

### 3.3. Source of Articles

All papers were published in peer reviewed journals. Publishers of the included studies are reported in Table 2. The journals with the highest number of published articles were Psychiatry Research (*n* = 2; 13.3%), Frontiers in Psychology (*n* = 2; 13.3%), and International Journal of Environmental Research and Public Health (*n* = 2; 13.3%). We also observed a global general interest in the psychological impact of the COVID-19 outbreak among young people, mainly involving Italian (*n* = 3; 20%), Chinese (*n* = 2; 13.3%), Malaysian (*n* = 2; 13.3%), and Bangladeshi (*n* = 2; 13.3%) researchers. Specifically, eight studies were conducted in Asia [26,27,29,31,32,33,36,37], five in Europe [24,25,28,35,38], and two in Africa [30,34]. In this narrative review, the adoption of restrictive inclusion criteria on the target population (general young population) generated the exclusion of American studies focused mostly on tailored target in the ongoing pandemic (see screening step in the flowchart above): the search strategy provided American studies conducted on vulnerable/fragile youth as well on young with previous mental health and/or stratified for race. 

Last, we observed that all first author’s institutions were universities.

### 3.4. Type of Articles 

Moreover, Table 3 highlights the details of screened articles that were grouped into cross-sectional and longitudinal studies. We extracted from each included paper: (a) study design, (b) topic, (c) measures, and (d) outcomes. All the papers focus on the impact of COVID-19 on mental health, while outcome tools mainly measured an increased risk of psychological diseases such as anxiety, depression, general distress, event-specific distress, and changes in sleep patterns.

### 3.5. Overview of Early Psychological Side Effects of COVID-19 for Young

First, this narrative review aimed to investigate the psychological impact of the COVID-19 outbreak among young people. Second, we analyzed the factors significantly associated with the prevalence of psychological symptoms.

Outcomes of the included studies showed that youth experienced anxiety [25,26,27,28,29,31,32,33,34,35,36,37,38], stress [33,34,37], depression [25,31,33,34,35,37], event-specific distress [33], and changes in sleep pattern [24] during the COVID-19 outbreak. One study [30] also underlined a decrease in perceived psychological wellbeing because of the lockdown. Moreover, higher-than-normal levels in somatization, obsessive-compulsive disorder, phobic anxiety, and paranoid ideation were found [32]. Cross-sectional studies [26,27,28,29,30,31,32,33,34,35,36,37] also explored how the COVID-19 pandemic influences the prevalence of psychological symptoms. Furthermore, with regard to the longitudinal studies included, two of these assessed and monitored the mental health of the young at two time points: before any restriction measures and during lockdown conditions [24,38]. One monitored the mental health status during the first month of lockdown during four time intervals [25]. Last, the psychological measures applied in examined studies were standardized tests addressed to the assessment of emotional dimensions (i.e., anxiety, depression, anger); only two studies performed semistructured interviews [24,29,30,33].

An analysis of the psychological symptomatology related to the COVID-19 outbreak is proposed below.

### 3.6. Anxiety

The findings we reviewed revealed an increased risk of anxiety during the COVID-19 outbreak and lockdown conditions for the young population [25,26,27,28,29,31,32,33,34,35,36,37,38]. A longitudinal study [38] also reported that individuals’ proneness to worry before the COVID-19 outbreak proved an impact on anxiety responses to the quarantine. High worriers at pre-lockdown showed, during lockdown conditions, a significant increase in anxiety and fear in terms of mental health in comparison to low worriers. Young people who were high worriers were more anxious and lower locus of control [38]. Similarly, a longitudinal study [25] comparing the Internalising and Externalising domains of the ASR/18–59, showed an analogous increase in the levels of anxiety while the lockdown measures were in place. The results of the included studies indicated that many factors were associated with psychological symptoms during the COVID-19 crisis. Female gender was associated with a higher level of anxiety during the COVID-19 pandemic compared to male [27,28,35,36]. This result is in contrast to what is reported from another study, indicating that males and females experienced similar levels of psychological symptoms as a result of the pandemic [26]. Research also underlines that levels of anxiety were significantly different according to age. Younger individuals experienced more anxiety compared to older ones [35,36,37].

Moreover, among the learning conditions, challenges of remote learning, the delay of final examinations, uncertainty related to exam dates, and concern about their academic performance were found to be risk factors for anxiety symptoms [26,27,28,29,31,32,36]. Healthcare and medical students had a lower risk of developing anxiety compared with students in other fields of study [36].

Among living conditions and place of residence during the lockdown, young people from urban areas experienced higher levels of anxiety compared to those living in rural areas [34]. In addition, tensions and conflict with family or occupants of the dwelling, difficulties isolating themselves, noisy environments, and no direct outside access through a garden, a terrace, or a balcony were associated with higher levels of anxiety [28]. Anxiety was also more prevalent among young people who did not perform physical activity [34] compared to young people who practiced physical exercise. Moreover, substance use and increased tobacco consumption as a coping strategy were risk factors for anxiety [28,34]. Finally, regarding strategies for managing anxiety, a study [29] interestingly reported that some students were unable to manage it: they reported practicing spiritual coping, following religious beliefs, and/or crying to vent their emotions. Counseling services seemed useful for helping them.

### 3.7. Depression

The results of the included studies also revealed that young people experienced depression symptomatology during the COVID-19 outbreak [25,31,33,34,35,37]. A longitudinal study [25] comparing the Internalising and Externalising domains of the ASR/18–59 showed an increase in the levels of depression while the lockdown measures were in place. Research did not find significant differences between genders except a study underlining that females experienced higher rates of emotional problems and depressive symptoms than males [35]. Moreover, according to age, the youngest experienced a higher level of depression compared to the oldest [35,37].

Among the learning conditions, most of the young were becoming depressed due to concern about their academic performance and the forced termination of their internships [31]. Regarding the field of study, students who engaged in health-science-related studies had less risk of developing depression compared with students in other fields of studies [34].

Among living conditions and place of residence during the lockdown, young people from urban areas had higher levels of depression compared to those living in rural areas [34]. Furthermore, young people who performed physical activity had a lower risk of developing depression [33,34] than young people who did not practice physical exercise. In addition, excessive exposure to COVID-19 news in social and mass media showed a significant association with higher scores in the DASS depression subscale [33].

### 3.8. Stress

Some studies also investigated psychological distress and revealed that young people experienced stress because of the COVID-19 outbreak and lockdown conditions [33,34,37]. Among the aspects influencing the prevalence of stress related to the COVID-19 pandemic, a study found that younger people experienced more stress than older people [37]. Regarding living conditions and place of residence, young people from urban areas and living with their families had higher levels of stress compared to those living in rural areas [34]. Furthermore, young who did not perform physical activity were at a higher risk of developing stress compared with students who did [34]. Furthermore, young people reported COVID-19-related social stressors such as financial uncertainty, fear of infection, inadequate food supply, lack of information on COVID-19, and excessive exposure to COVID-19 news in social and mass media [33].

### 3.9. Event-Specific Distress

Only one study of the included papers investigated the prevalence of event-specific distress caused by the COVID-19 pandemic. This study [33] observed that 69.31% of respondents had event-specific distress caused by the outbreak from mild to severe, according to IES. Specifically, fear of infection, perceived social media as a stressor, and inadequate valid information on COVID-19 had a significant association with higher scores on the IES scale. Furthermore, among the socio-demographic characteristics, results indicated that older students scored higher on IES [33].

### 3.10. Psychological Wellbeing

Regarding, general psychological wellbeing, one study [30] underlined that 55% of the respondents reported decreased psychological wellbeing because of the lockdown. Digital skills, as well as technological advances, seemed to be a positive factor dealing with the social restrictive measures in the pandemic. Among students, online chatting with friends, watching films, and focusing on online capacity developments were identified as protective coping strategies against the deterioration of mental health during the lockdown. Meanwhile, craving for substances as a coping strategy and lack of satisfaction with the online mode of teaching were significant predictors of decreased psychological wellbeing among the respondents [30].

### 3.11. Quality of Sleep

The COVID-19 outbreak, lockdown restrictions, and extraordinary measures to curb the spread of coronavirus also influenced changes in sleep habits. One study [24] observed a significant increase in the PSQI score under the restriction. The PSQI is a self-rated questionnaire that assesses sleep quality and disturbances over a one-month time interval [39]. Specifically, sleep–wake rhythms markedly changed under restriction. Young people went to bed and woke up later and spend more time in bed. However, paradoxically, they also reported lower sleep quality. In addition, the decrease in sleep quality was stronger for people with a higher level of anxiety, stress, and depression symptomatology [24].

### 3.12. Other Changes in Mental Health Status

Concerning other changes in mental health status, a longitudinal study [25] comparing the Internalising and Externalising domains of the ASR/18–59 showed an analogous increase for both areas from the first to the fourth week of lockdown. The ASR is a self-report questionnaire for ages 18-59 that assesses behavioral, emotional, and social problems; adaptive functioning; personal strength; and substance use [40]. Specifically, the levels of Withdrawal and Somatic Complaints (Internalising problems area) and the levels of Aggressive Behaviour and Rule Breaking Behaviour (Externalising problems area) overall increased while the lockdown measures were in place [25]. Moreover, one study [32] underlined higher-than-normal levels of somatization, obsessive-compulsive disorder, interpersonal sensitivity, phobic anxiety, and paranoid ideation. This indicates that the mental health status of university students was clearly worsened.

### 3.13. Stressors and Protective Factors

In addition to the result mentioned above, having relatives or friends infected by COVID-19 [26,28,34], financial uncertainty [26,31,33,36], worsening of interpersonal conflict, and restriction in social contact [35] were identified as the most prominent stressors. On the other hand, specific protective factors can be detected in family, friendship, and social support [26,28]; social activities such as spending time with family members, online chatting with friends, online and offline gaming, watching TV, reading storybooks [29,30,33]; having a steady family income [26]; and setting up regular schedules and routines in daily life in terms of work, eating, leisure time, exercising, and sleep [35].

## 4. Discussion

The COVID-19 outbreak has globally affected and is still affecting not only physical health but also mental health and wellbeing [5]. Considering emerging scientific highlights on the effects of COVID-19 on youth’s wellbeing [41,42], this narrative review focused on the impact of the COVID-19 outbreak, specifically on the mental health of the young population. We also explored factors influencing the prevalence of psychological symptoms related to the COVID-19 pandemic. Concerning the pandemic, social restrictions, and extraordinary measures to contain the spread of the coronavirus, almost all of the studies that were considered by this review collected data through online survey, showing the strong and positive impact of digital solutions. In general, the survey links were disseminated on various platforms available on social media (e.g., Facebook, WhatsApp, email invitation, Google Forms). Regarding mental health and wellbeing, the most analyzed variables were anxiety, mental distress, and depression. Other analyzed variables included event-specific distress, quality of sleep, and psychological wellbeing. Although the lockdown measures reduced the spread of COVID-19 infection, they had several side effects. Included studies showed that the young people frequently experienced anxiety [25,26,27,28,29,31,32,33,34,35,36,37,38], mental distress [33,34,37], depression [25,31,33,34,35,37], event-specific distress [33], changes in sleep pattern [24], decrease in psychological wellbeing [30], and higher-than-normal levels in somatization, obsessive-compulsive disorder, phobic anxiety, and paranoid ideation [32] during the COVID-19 pandemic.

These findings also revealed that several factors were associated with psychological symptoms related to the COVID-19 outbreak. Socio-demographic characteristics, gender, age, and living conditions were significantly associated with mental health. Female gender was associated with a higher level of anxiety and depression during the COVID-19 outbreak than the male one [27,28,35,36]. On the contrary, one study [26] reported no gender effect on negative emotional reaction to pandemic measures. In any case, most studies indicate that females show higher psychological symptoms, such as anxiety and depression, than males [43]. Furthermore, younger people experienced high levels of anxiety, stress, and depression compared to older ones [35,36,37]. Age-related differences may be linked to less resilience in the adaption to changes [44].

Among living conditions during the lockdown, the results indicated that many factors were associated with psychological symptoms during the COVID-19 crisis. Specifically, regarding the place of residence, young people from urban areas had higher levels of anxiety, stress, and depression compared to those living in rural areas [34]. This might be due to the higher prevalence of COVID-19 in urban residences than rural areas and due to the difficulty in implementing physical distancing due to the condensed population. In addition, tensions and conflict with family, noisy environments, and no easy access to outdoor activities were associated with higher levels of anxiety [28].

Among the factors that were associated with psychological symptoms related to the COVID-19 outbreak, we also explored higher education. As expected, universities adopted massive e-learning. Students’ lives drastically changed, and reducing their social interactions and leaving them dealing with obstacles for new technological and digital learning settings. All these changes affected the mental health of young people. Among the learning condition, challenges of remote learning, the delay of final exams, uncertainty related to exam dates, and general concern about their academic performance were found to be risk factors for anxiety symptoms, depression, and deterioration of psychological wellbeing [26,27,28,29,30,31,32,36]. Regarding study program, healthcare and medical students had a lower risk of developing anxiety and depression compared with students in other programs [34,36]. This might be due to the fact that healthcare students might have been more well-informed about the effects of the pandemic compared to other students.

Performing physical activity was associated with a lower risk of developing anxiety, stress, and depression [33,34] compared to a more sedentary life. These findings are consistent with a previous study that suggests physical activity to have a positive effect on mental health [45].

Risk for substance use was a significant predictor for decreased psychological wellbeing and higher levels of anxiety [28,30,34]. The reason might be due to psychological or physiological dependence following substance use. Substance use might cause or worsen other life problems and might ultimately worsen anxiety [34].

Excessive exposure to COVID-19 news in social and mass media had a significant association with depression, stress, and psychological impact in terms of event-specific distress [33]. The COVID-19 outbreak, the lockdown restrictions, and the extraordinary measures to curb the spread of coronavirus also influenced the changes in sleep habits. Young people reported deterioration of sleep quality under restriction; this was stronger for people with a higher level of anxiety, stress, and depression symptomatology [24]. Furthermore, findings indicated a high rate of obsessive-compulsive disorders [32], mostly with regard to the extensive suggestion about handwashing as a preventive action for COVID-19 infection. In general, the worsened psychological status might be due to the specific circumstances during the COVID-19 outbreak such as lockdown condition, social restriction, fear of contamination, challenges of e-learning, uncertainty about the pandemic progress, and more. Last, psychological difficulties were related to the experience of several domains of stressors, such as financial uncertainty [26,31,33,36], having relatives or friends infected by COVID-19 [26,28,34], and social restriction [35].

Specific protective factors can be detected in family, friendship, and social support [26,28] as well as spending time with family members, online chatting with friends, online and offline gaming, watching TV, and reading storybooks [29,30,33]. The available scientific literature related to mental health aspects of the young impacted by COVID-19 outbreak and lockdown, highlights a need to develop strategies and interventions against psychological consequences caused by COVID-19 pandemic.

## 5. Conclusions

The COVID-19 outbreak has globally affected and is still affecting youth mental health. This narrative review indicates a significant impact of the pandemic and the lockdown measures for several mental symptoms including anxiety, mental distress, depression, psychological wellbeing, and sleep habits of young people. The psychological impact was related to the experience of several intensive stressors, such as academic perspectives, massive e-learning adoption, economic obstacles, social restrictions, and daily living side effects related to the COVID-19 outbreak. This narrative review highlighted the relevance of focusing on preventive and strategic actions on mental health for young people during the pandemic and the urgent need afterward for psychological and supportive interventions. Youth mental health actions should be the priority and challenge for drawing strategic plans in the future: (a) to determine and implement desired mental health consultations; (b) to develop additional resources for direct mental health service to high-need youth; (c) to maximize peer support and exchange of ideas; (d) to increase the level of cultural competency of mental health services and approach; and (e) to create more within-program resources for mental health.

This narrative review has some limits. One limitation to the present review is that almost all of the studies included collected data through online survey. Online surveys allow one to assess the prevalence of psychological symptoms related to the COVID-19 outbreak in young people while preserving the social distance and all the extraordinary measures to curb the spread of coronavirus. However, the use of electronic self-report questionnaires may have excluded people without internet access, and although anonymous, the study may not be totally free from self-reporting bias. Almost all the included research papers were cross-sectional studies not reporting information about the participant’s psychological symptoms before the pandemic.

## Figures and Tables

**Table 1:** Main characteristics of the included studies.

Study Design	Authors	Sample Size	Age Range	Sample	Data Collection Timing	Recruitment
Cross-sectional study	Baloch et al. (2021) [27]	*n* = 494	below 18 years (9.1%) 19–25 (77.3%) above 26 (13.5%)	College and university students	From 26 May to 6 June 2020	Online survey (WhatsApp, Email)
	Bourion-Bédès et al. (2021) [28]	*n* = 3928	Average = 21.74 ± 4	College and University students	May, 2020	Online survey
	Cao et al. (2020) [26]	*n* = 7143	Undergraduate age	College students	-	Not mentioned
	Faize & Husain (2021) [29]	*n* = 342	Undergraduate age	University students	-	Online survey
	Idowu et al. (2020) [30]	*n* = 433	15–40 Average = 20.11 ± 2.9	University students	April and May 2020	Online survey
	Islam et al. (2020) [31]	*n* = 476	17 and older	University students	March, 2020	Online survey (Google Forms)
	Jiang (2020) [32]	*n* = 472	17–22	University students	Febrary 2020	Online survey (Star software platform)
	Khan et al. (2020) [33]	*n* = 505	19 or less (12.67%) 20–24 (78.42%) 25 or more (8.91%)	College and university students	April 2020	Online survey on social media (Facebook)
	Mekonen et al. (2021) [34]	*n* = 350	20 and older	University students	November 2020	Graduating class students available during the data collection period
	Padrón et al. (2021) [35]	*n* = 932	18 and older	University students	-	Online survey (internal web application)
	Sundarasen et al. (2020) [36]	*n* = 983	17 and older	University students	From 20 April to 24 May 2020	Online survey
	Wan Mohd Yunus et al. (2020) [37]	*n* = 1005	17 and older	University students	April 2020	Online survey (Qualtrics survey platform)
Longitudinal study	Baiano et al. (2020) [38]	*n* = 25	Average = 23.84 ± 2.5	University students	April 2020	Online survey (Google Forms)
	Cellini et al. (2020) [24]	*n* = 1310	18–35 Average = 23.91 ± 3.6	University students Young workers	March 2020	Online survey
	Parola et al. (2020) [25]	*n* = 97	19–29	Young in a lockdown condition	March-April 2020	Online survey on social media

**Table 2:** Publishers of studies included in the present narrative review.

		Number	Ratio (%)
**Source Title**	Psychiatry Research	2	13.3
	Frontiers in Psychology	2	13.3
	International Journal of Environmental Research and Public Health	2	13.3
	Journal of Affective Disorders	1	6.7
	Children and Youth Services Review	1	6.7
	Peer J	1	6.7
	Psychology Research and Behaviour Management	1	6.7
	Frontiers in Psychiatry	1	6.7
	Journal of Medical and Surgical Research	1	6.7
	Journal of Mental Health Training Education and Practice	1	6.7
	Journal of Sleep Research	1	6.7
	PLoS One	1	6.7
**Continent of research**	Asia	8	53.3
	Europe	5	33.3
	Africa	2	13.3
**First author’s country**	Italy	3	20
	China	2	13.3
	Malaysia	2	13.3
	Bangladesh	2	13.3
	Pakistan	1	6.7
	France	1	6.7
	Ethiopia	1	6.7
	Spain	1	6.7
	Saudi Arabia	1	6.7
	Nigeria	1	6.7
**First author’s institution**	University	15	100

**Table 3:** Summary of included studies evaluating the psychological impact of COVID-19 among young people.

Study Design	Authors	Topic	Measures	Outcomes
Cross-sectional study	Baloch et al. (2021) [27]	Impact of COVID-19 on mental health	▪ Zung SAS	The results indicate that approximately 41% of the respondents experienced minimal to moderate, marked to severe, and most extreme levels of anxiety. Female students were more anxious than male ones. The most prominent stressors are associated with online teaching, concerns about their academic performance and completion of the current semester, uncertainty related to exam dates, and the status of the following semester.
	Bourion-Bédès et al. (2021) [28]	Impact of COVID-19 on mental health	▪ GAD-7 ▪ MSPSS	Sixty-one percent of students in the sample experienced anxiety during the lockdown due to the COVID-19 outbreak. Female gender, having relatives infected with COVID-19, conflicts at home, difficulties isolating themselves, noisy environments, no direct outside access, delay in final examinations, reduced time for learning, and increased tobacco consumption were the main risk factors for anxiety. Support from family and friends was a protective factor.
	Cao et al. (2020) [26]	Impact of COVID-19 on mental health	▪ GAD-7	About 24.9% of respondents experienced anxiety because of the COVID-19 outbreak. Family income stability, living with parents and social support were protective factors against anxiety. Gaving relatives infected with COVID-19, economic stressors, academic delay, and effects on daily life were positively associated with anxiety symptoms.
	Faize and Husain (2021) [29]	Impact of COVID-19 on mental health	▪ GAD-7 ▪ Semi-structured interview conducted with students diagnosed with severe anxiety through GAD-7	Among the respondents, 21.6% had mild, 9.4% had moderate and only 8.2% had severe anxiety. Students with severe anxiety reported psychological, social, and physical problems related to COVID-19, during the interview. Students managed their anxiety engaging in different personal activities at home, adopting COVID-19 safety guidelines, and avoiding media. Moreover, some students reported their inability to cope with their problems.
	Idowu et al. (2020) [30]	Impact of COVID-19 on psychological wellbeing	▪ Self-administered, semi-structured questionnaire collecting information on student life during the pandemic, the psychological impact of COVID 19, and their coping strategies	A total of 55.0% of the respondents decreased psychological wellbeing because of the lockdown. Protective factors included online chatting with friends, watching films, and focusing on online capacity development. Meanwhile, craving for substances as a coping strategy and lack of satisfaction with the online mode of teaching were the significant predictors of decreased psychological wellbeing.
	Islam et al. (2020) [31]	Impact of COVID-19 on mental health	▪ PHQ-9 ▪ GAD-7	The findings indicate that more than two-thirds of the students were experiencing mild to severe depression (82.4%) and anxiety (87.7%), suggesting that university students are experiencing an unparalleled growth in depression and anxiety under the global pandemic situation. The prolonged unemployment, together with financial insecurity and concern about their academic performance were the most significant stressors contributing to the increased rates of depression and anxiety.
	Jiang (2020) [32]	Impact of COVID-19 on mental health	▪ SCL-90 ▪ COVID-19 General Information Questionnaire	The results revealed higher than normal levels in somatization, obsessive-compulsive disorder, interpersonal sensitivity, anxiety, phobic anxiety, paranoid ideation, and general severity index, during the pandemic, indicating that the mental health status of university students was clearly worse than the norm.
	Khan et al. (2020) [33]	Impact of COVID-19 on mental health and wellbeing	▪ DASS-21 ▪ IES ▪ Self-reported physical symptoms ▪ Home-quarantine activities ▪ COVID-19 related social stressors perceived as psychological discomfort	In total, 28.5% of the respondents experienced stress, 33.3% anxiety, and 46.92% depression from mild to extremely severe, according to DASS-21. A total of 69.31% had event-specific distress from mild to severe according to IES. Specifically, older students had a higher psychological impact in terms of IES scores than younger students. Perceiving physical symptoms similar to the symptoms of COVID-19, fear of infection, financial uncertainty, inadequate food supply, no physical activity, and limited or no social activity had a significant association with stress, anxiety, depression, and post-traumatic symptoms. Excessive exposure to COVID-19 news in social and mass media had a significant association with depression, stress, and psychological impact in terms of event-specific distress. Contrary, students involved with various activities like physical exercise, recreational activity (watching TV series, reading storybooks, online, and offline gaming), and household chores have coped with the situation better.
	Mekonen et al. (2021) [34]	Impact of COVID-19 on mental health	▪ DASS-21	The prevalence of stress, anxiety, and depression among graduating class students was 22.2%, 39.6%, and 40.2%, respectively. Living in an urban area, sedentary lifestyle, unable to practice COVID-19 preventive measures, and having a contact history increased the risk of developing stress. Living in an urban area, substance use, sedentary lifestyle, and fear of infecting family increased the risk of developing anxiety. Students who came from an urban area, studied non-health departments, had confirmed cases in the family, and did not perform physical exercise had higher odds of developing depression.
	Padrón et al. (2021) [35]	Impact of COVID-19 on mental health	▪ GAD-7 ▪ PHQ-9 ▪ BIT ▪ Self-perceived change in mental health	Results indicated that students experienced considerable psychological problems during the lockdown, with higher rates of emotional difficulties in female and younger students than in male and older students, respectively. Psychological distress was mainly related to academic future, worsening of interpersonal conflicts, and restrictions in social contact. As regards coping strategies, both reframing skills and daily routines (work, leisure time, exercising, and sleeping) mediated the effects of stressors on psychological health.
	Sundarasen et al. (2020) [36]	Impact of COVID-19 on mental health	▪ Zung SAS	In total, 20.4%, 6.6%, and 2.8% of respondents experienced minimal to moderate, marked to severe, and most extreme levels of anxiety. Female gender, younger age, pre-university level of education, management studies, and staying alone were significantly associated with higher levels of anxiety. Furthermore, stressors are predominantly related to financial constraints, remote online learning, uncertainty on academic performance, and future career prospects.
	Wan Mohd Yunus et al. (2020) [37]	Impact of COVID-19 on mental health	▪ DASS-21 ▪ OHQ ▪ WFC	In total, 22%, 34.3%, and 37.3% of the university students scored moderate to extremely severe levels of stress, anxiety, and depression symptoms, respectively. The level of stress, anxiety, and depression were significantly different according to age: younger students experienced more stress, anxiety, and depression symptoms compared with older ones.
Longitudinal study	Baiano et al. (2020) [38]	Impact of COVID-19 on mental health	▪ PSWQ ▪ ASI-3 ▪ MAAS	Individual’s proneness to worry before the COVID-19 outbreak proved to have an impact on anxiety responses to the quarantine. High worriers at pre-lockdown showed, during lockdown conditions, a significant increase in anxiety sensitivity and fear of mental health in comparison to low worriers: high worriers were more anxious and specifically concerned about the mental correlates of anxiety symptoms considered signals of cognitive decontrol.
	Cellini et al. (2020) [24]	Impact of COVID-19 on sleep patterns and mental health	▪ DASS-21 ▪ PSQI ▪ Questionnaire on digital media use near bedtime ▪ Questionnaire on experience time	During home confinement, sleep–wake rhythms markedly changed, with people going to bed and waking up later, and spending more time in bed, but paradoxically also reporting a lower sleep quality. The decrease in sleep quality was stronger for people with a higher level of depression, anxiety, and stress symptomatology.
	Parola et al. (2020) [25]	Impact of COVID-19 on mental health	▪ ASR/18–59	Comparing the Internalising and Externalising domains of the ASR/18–59, the results showed an analogous increase for both areas from the first to the fourth week of lockdown restrictions. Specifically, the levels of Anxiety/Depression, Withdrawal, and Somatic Complaints (Internalising problems area) and the levels of Aggressive Behaviour and Rule Breaking Behaviour (Externalising problems area) overall increased while the lockdown measures were in place.

Abbreviation of measures: Depression Anxiety Stress Scale (DASS-21) [33,34,37], Zung Self-Rating Anxiety Scale (SAS) [27,36], General Anxiety Disorder-7 (GAD-7) [26,28,29,31,35], Patient Health Questionnaire (PHQ-9) [31,35], Impact of Event Scale (IES) [33], 90-item Symptom Checklist (SCL-90) [32], Multidimensional Scale of Perceived Social Support (MSPSS) [28], Oxford Happiness Questionnaire (OHQ) [37], Work-family Conflict Scale (WFC) [37], Brief Irritability Test (BIT) [35], Adult Self-Report (ASR/18–59) [25], Penn State Worry Questionnaire (PSWQ) [38], Anxiety Sensitivity Index-3 (ASI-3) [38], Mindful Attention Awareness Scale (MAAS) [38], Pittsburgh Sleep Quality Index (PSQI) [24]. In addition to these standardised psychological tests, authors also used ad hoc questionnaires [24,30,32,33,35] and interviews [29].

## References

[1] Zhu N., Zhang D., Wang W., Li X., Yang B., Song J., Zhao X., Huang B., Shi W., Lu R. (2020). A Novel Coronavirus from Patients with Pneumonia in China, 2019. N. Engl. J. Med..

[2] World Health Organization WHO Director-General’s opening remarks at the media briefing on COVID-19—11 March 2020. https://www.who.int/director-general/speeches/detail/who-director-general-s-opening-remarks-at-the-media-briefing-on-covid-19---11-march-2020.

[3] Umakanthan S., Sahu P., Ranade A.V., Bukelo M.M., Rao J.S., Abrahao-Machado L.F., Dahal S., Kumar H., Kv D. (2020). Origin, transmission, diagnosis and management of coronavirus disease 2019 (COVID-19). Postgrad. Med. J..

[4] Shrestha N., Shad M.Y., Ulvi O., Khan M.H., Karamehic-Muratovic A., Nguyen U-SD T., Baghbanzadeh M., Wardrup R., Aghamohammadi N., Cervantes D. (2020). The impact of COVID-19 on globalization. One Health.

[5] Fiorillo A., Gorwood P. (2020). The consequences of the COVID-19 pandemic on mental health and implications for clinical practice. Eur. Psychiatry.

[6] Jia N., Fan N., Lu Z. (2003). A survey of the undergraduate anxiety in the SARS-infected areas. J. Hebei Norm. Univ..

[7] Maunder R., Hunter J., Vincent L., Bennett J., Peladeau N., Leszcz M., Sadavoy J., Verhaeghe L.M., Steinberg R., Mazzulli T. (2003). The immediate psychological and occupational impact of the 2003 SARS outbreak in a teaching hospital. CMAJ.

[8] Tam C.W.C., Pang E.P.F., Lam L.C.W., Chiu H.F.K. (2004). Severe acute respiratory syndrome (SARS) in Hong Kong in 2003: Stress and psychological impact among frontline healthcare workers. Psychol. Med..

[9] Hawryluck L., Gold W.L., Robinson S., Pogorski S., Galea S., Styra R. (2004). SARS control and psychological effects of quarantine, Toronto, Canada. Emerg. Infect. Dis..

[10] Liu X., Kakade M., Fuller C.J., Fan B., Fang Y., Kong J., Guan Z., Wu P. (2012). Depression after exposure to stressful events: Lessons learned from the severe acute respiratory syndrome epidemic. Compr. Psychiatry.

[11] Wheaton M.G., Abramowitz J.S., Berman N.C., Fabricant L.E., Olatunji B.O. (2012). Psychological Predictors of Anxiety in Response to the H1N1 (Swine Flu) Pandemic. Cognit. Ther. Res..

[12] Taylor M.R., Agho K.E., Stevens G.J., Raphael B. (2008). Factors influencing psychological distress during a disease epidemic: Data from Australia’s first outbreak of equine influenza. BMC Public Health.

[13] Jalloh M.F., Li W., Bunnell R.E., Ethier K.A., O’Leary A., Hageman K.M., Sengeh P., Jalloh M.B., Morgan O., Hersey S. (2018). Impact of Ebola experiences and risk perceptions on mental health in Sierra Leone, July 2015. BMJ Glob. Health.

[14] Rossi R., Socci V., Talevi D., Mensi S., Niolu C., Pacitti F., Di Marco A., Rossi A., Siracusano A., Di Lorenzo G. (2020). COVID-19 pandemic and lockdown measures impact on mental health among the general population in Italy. Front. Psychiatry.

[15] Kang L., Ma S., Chen M., Yang J., Wang Y., Li R., Yao L., Bai H., Cai Z., Xiang Yang B. (2020). Impact on mental health and perceptions of psychological care among medical and nursing staff in Wuhan during the 2019 novel coronavirus disease outbreak: A cross-sectional study. Brain Behav. Immun..

[16] Merlo M.E., Sicari F., Frisone F., Costa G., Alibrandi A., Avena G., Settineri S. (2021). Uncertainty, alexithymia, suppression and vulnerability during the COVID-19 pandemic in Italy. Health Psychol. Rep..

[17] Ranieri J., Guerra F., Di Giacomo D. (2021). Predictive risk factors for post-traumatic stress symptoms among nurses during the Italian acute COVID-19 outbreak. Health Psychol. Rep..

[18] Wilczyńska D., Li J., Yang Y., Fan H., Liu T., Lipowski M. (2021). Fear of COVID-19 changes the motivation for physical activity participation: Polish-Chinese comparisons. Health Psychol. Rep..

[19] Zhou S.J., Zhang L.G., Wang L.L., Guo Z.C., Wang J.Q., Chen J.C., Liu M., Chen X., Chen J.X. (2020). Prevalence and socio-demographic correlates of psychological health problems in Chinese adolescents during the outbreak of COVID-19. Eur. Child Adolesc. Psychiatry.

[20] Fegert J.M., Vitiello B., Plener P.L., Clemens V. (2020). Challenges and burden of the Coronavirus 2019 (COVID-19) pandemic for child and adolescent mental health: A narrative review to highlight clinical and research needs in the acute phase and the long return to normality. Child Adolesc. Psychiatry Ment. Health.

[21] Ravens-Sieberer U., Kaman A., Erhart M., Devine J., Schlack R., Otto C. (2021). Impact of the COVID-19 pandemic on quality of life and mental health in children and adolescents in Germany. Eur. Child Adolesc. Psychiatry.

[22] Arksey H., O’Malley L. (2005). Scoping studies: Towards a methodological framework. Int. J. Soc. Res. Methodol..

[23] Moher D., Liberati A., Tetzlaff J., Altman D.G., Group T.P. (2009). Preferred Reporting Items for Systematic Reviews and Meta-Analyses: The PRISMA Statement. PLoS Med..

[24] Cellini N., Canale N., Mioni G., Costa S. (2020). Changes in sleep pattern, sense of time and digital media use during COVID-19 lockdown in Italy. J. Sleep Res..

[25] Parola A., Rossi A., Tessitore F., Troisi G., Mannarini S. (2020). Mental Health Through the COVID-19 Quarantine: A Growth Curve Analysis on Italian Young Adults. Front. Psychol..

[26] Cao W., Fang Z., Hou G., Han M., Xu X., Dong J., Zheng J. (2020). The psychological impact of the COVID-19 epidemic on college students in China. Psychiatry Res..

[27] Baloch G.M., Sundarasen S., Chinna K., Nurunnabi M., Kamaludin K., Khoshaim H.B., Hossain S.F.A., AlSukayt A. (2021). COVID-19: Exploring impacts of the pandemic and lockdown on mental health of Pakistani students. PeerJ.

[28] Bourion-Bédès S., Tarquinio C., Batt M., Tarquinio P., Lebreuilly R., Sorsana C., Legrand K., Rousseau H., Baumann C. (2021). Psychological impact of the COVID-19 outbreak on students in a French region severely affected by the disease: Results of the PIMS-CoV 19 study. Psychiatry Res..

[29] Faize F.A., Husain W. (2021). Students with severe anxiety during COVID-19 lockdown—Exploring the impact and its management. J. Ment. Health Train. Educ. Pract..

[30] Idowu A., Olawuyi D.A., Nwadioke C.O. (2020). Impacts of covid-19 pandemic on the psychological wellbeing of students in a Nigerian university. JMSR.

[31] Islam M.A., Barna S.D., Raihan H., Khan M.N.A., Hossain M.T. (2020). Depression and anxiety among university students during the COVID-19 pandemic in Bangladesh: A web-based cross-sectional survey. PLoS ONE.

[32] Jiang R. (2020). Knowledge, attitudes and mental health of university students during the COVID-19 pandemic in China. Child. Youth Serv. Rev..

[33] Khan A.H., Sultana M.S., Hossain S., Hasan M.T., Ahmed H.U., Sikder M.T. (2020). The impact of COVID-19 pandemic on mental health & wellbeing among home-quarantined Bangladeshi students: A cross-sectional pilot study. J. Affect. Disord..

[34] Mekonen E.G., Workneh B.S., Ali M.S., Muluneh N.Y. (2021). The Psychological Impact of COVID-19 Pandemic on Graduating Class Students at the University of Gondar, Northwest Ethiopia. Psychol. Res. Behav. Manag..

[35] Padrón I., Fraga I., Vieitez L., Montes C., Romero E. (2021). A Study on the Psychological Wound of COVID-19 in University Students. Front. Psychol..

[36] Sundarasen S., Chinna K., Kamaludin K., Nurunnabi M., Baloch G.M., Khoshaim H.B., Hossain S.F.A., Sukayt A. (2020). Psychological Impact of COVID-19 and Lockdown among University Students in Malaysia: Implications and Policy Recommendations. Int. J. Environ. Res. Public Health.

[37] Wan Mohd Yunus W.M.A., Badri S.K.Z., Panatik S.A., Mukhtar F. (2020). The Unprecedented Movement Control Order (Lockdown) and Factors Associated with the Negative Emotional Symptoms, Happiness, and Work-Life Balance of Malaysian University Students During the Coronavirus Disease (COVID-19) Pandemic. Front. Psychiatry.

[38] Baiano C., Zappullo I., Group T.L., Conson M. (2020). Tendency to Worry and Fear of Mental Health during Italy’s COVID-19 Lockdown. Int. J. Environ. Res. Public Health.

[39] Buysse D.J., Reynolds C.F., Monk T.H., Berman S.R., Kupfer D.J. (1989). The Pittsburgh Sleep Quality Index: A new instrument for psychiatric practice and research. Psychiatry Res..

[40] Achenbach T.M., Rescorla L.A. (2003). Manual for the ASEBA Adult Forms & Profiles.

[41] Kjøs P., Klippen I., Hovgaard H., Krokstad S., Sletten M.A., Lekang B., Konar M., Møgster R.L., Antonsen M., Modalen M. (2021). Livskvalitet, psykisk helse og rusmiddelbruk under Covid-19-pandemien. [Life Quality, Mental Health, and Use of Substances during the Covid-19 Pandemic]. https://www.fhi.no/div/helseundersokelser/fylkeshelseundersokelser/livskvalitet-og-psykisk-helse-under-koronaepidemien--nov-des-2020/.

[42] Nøkleby H., Berg R.C., Muller A.E., Ames H.M.R. (2021). Konsekvenser av covid-19 på barn og unges liv og helse: En hurtigoversikt. [The Effects of Covid-19 on Children and Youth’s Wellbeing: A Rapid Review].

[43] Altemus M., Sarvaiya N., Neill Epperson C. (2014). Sex differences in anxiety and depression clinical perspectives. Front. Neuroendrocinol..

[44] Masten A.S., Obradović J., Burt K.B. (2006). Resilience in Emerging Adulthood: Developmental Perspectives on Continuity and Transformation. Emerging Adults in America: Coming of Age in the 21st Century.

[45] Peluso M.A.M., Guerra de Andrade L.H.S. (2005). Physical activity and mental health: The association between exercise and mood. Clinics (Sao Paulo).

